# *Staphylococcus aureus* Specific Electrospun Wound Dressings: Influence of Immobilization Technique on Antibacterial Efficiency of Novel Enzybiotic

**DOI:** 10.3390/pharmaceutics13050711

**Published:** 2021-05-13

**Authors:** Olga Urbanek, Alicja Wysocka, Paweł Nakielski, Filippo Pierini, Elżbieta Jagielska, Izabela Sabała

**Affiliations:** 1 International Institute of Molecular and Cell Biology in Warsaw, 02-109 Warsaw, Poland; ourbanek@ippt.pan.pl (O.U.); awysocka@iimcb.gov.pl (A.W.); pnakiel@ippt.pan.pl (P.N.); fpierini@ippt.pan.pl (F.P.); ejagielska@iimcb.gov.pl (E.J.); 2 Institute of Fundamental Technological Research, Polish Academy of Sciences, 02-106 Warsaw, Poland

**Keywords:** antibacterial wound dressings, enzybiotic, fibers functionalization, electrospun wound dressings, *Staphylococcus aureus*

## Abstract

The spread of antimicrobial resistance requires the development of novel strategies to combat superbugs. Bacteriolytic enzymes (enzybiotics) that selectively eliminate pathogenic bacteria, including resistant strains and biofilms, are attractive alternatives to antibiotics, also as a component of a new generation of antimicrobial wound dressings. Auresine*Plus* is a novel, engineered enzybiotic effective against *Staphylococcus aureus*—one of the most common pathogenic bacteria, found in infected wounds with a very high prevalence of antibiotic resistance. We took advantage of its potent lytic activity, selectivity, and safety to prepare a set of biodegradable PLGA/chitosan fibers generated by electrospinning. Our aim was to produce antimicrobial nonwovens to deliver enzybiotics directly to the infected wound and better control its release and activity. Three different methods of enzyme immobilization were tested: physical adsorption on the previously hydrolyzed surface, and covalent bonding formation using *N*-hydroxysuccinimide/*N*-(3-Dimethylaminopropyl)-*N*′-ethylcarbodiimide (NHS/EDC) or glutaraldehyde (GA). The supramolecular structure and functional properties analysis revealed that the selected methods resulted in significant development of nanofibers surface topography resulting in an efficient enzybiotic attachment. Both physically adsorbed and covalently bound enzymes (by NHS/EDC method) exhibited prominent antibacterial activity. Here, we present the extensive comparison between methods for the effective attachment of the enzybiotic to the electrospun nonwovens to generate biomaterials effective against antibiotic-resistant strains. Our intention was to present a comprehensive proof-of-concept study for future antimicrobial wound dressing development.

## 1. Introduction

*Staphylococcus aureus* (*S. aureus*) is one of the most common pathogenic bacteria isolated from infected wounds, and at the same time, one of the most difficult to treat [1,2]. This is due to a very high level of antibiotic resistance among *S. aureus* strains [3,4], and because of their tendency to generate biofilms and form persistent cells [1,5,6,7]. Antimicrobial wound management is a major challenge that continues to require new solutions against microbes and their biofilms. The main goal of antiseptic therapy is to eliminate bacteria to prevent infections. There are antibacterial agents combined with wound dressing that are available for treatment, like antibiotics, silver particles, hydrogen peroxide, and iodine-based preparation. Topical antibiotics can be very effective when used against sensitive organisms, yet in general, they are not recommended because of minimal effectiveness, potential contribution to the formation of resistant strains, and possible sensitization. Moreover, the currently used antibacterial treatments eliminate not only pathogenic bacteria, but also commensals, which had been shown to play an important role in wound healing [5]. Hence, more and more attention is being attributed to a selective treatment, eliminating only pathogenic bacterial species [5,6]. Enzybiotics seem to be a perfect component of the new generation, effective and selective wound dressings [7,8,9]. They are lytic enzymes of phage or bacterial origin, which exhibit very high efficiency in bacteria elimination, often limited to the pathogenic bacterial strains. Notably, the prevalence of resistance development against this class of antimicrobials is reported to be very low [10]. The therapeutic potential of enzybiotics has already been demonstrated in many examples, as summarized in the phiBIOTICS database [11].

In our current work, we exploit the potential of Auresine*Plus*, a chimeric enzyme that consists of the catalytic domain of peptidoglycan hydrolase LytM [12,13] fused to the cell wall targeting domain of lysostaphin [14], which demonstrates a unique antistaphylococcal activity [15]. Auresine*Plus* eradicates millions of bacterial cells of the antibiotic-resistant *S. aureus* in minutes. It targets bacteria both in planktonic and biofilm forms—the latter is the microbial consortia commonly found in the chronic wounds [2]. The high lytic efficiency and specificity towards *S. aureus*, superior stability, and no cytotoxicity, and well-developed production technology make Auresine*Plus*, a perfect enzybiotic, ready-to-use in the novel antibacterial therapy, particularly as a component of the wound dressings.

An ideal wound dressing should sufficiently absorb the exudates from the wound surface and induce cell proliferation to facilitate regeneration and at the same time eliminate pathogenic bacteria [16]. One of the most advanced wounds dressings are nonwovens formed via electrospinning [17]. Electrospun fiber networks mimic the native extracellular matrix (ECM), creating an environment suitable for cell growth [18]. By applying post-treatment modifications, fiber surfaces can be functionalized to achieve unique properties, among them antimicrobial activity [19,20]. High surface-to-volume ratio and interconnected pores provide an extended surface for attaching bioactive molecules or particles, which secures good gas exchange and nutrient supply [21]. Though various methods can be used to fabricate biomaterials, we have focused on electrospinning that generates fibers with desired features, that we successfully used previously to generate various nonwovens for medical applications [22,23].

To form nanofibers with antibacterial capabilities, their surface has been functionalized with, e.g., various antimicrobial chemotherapeutics (e.g., sulfadiazine, ciprofloxacin, tetracycline), nanoparticles (e.g., silver, zinc oxide, titanium oxide), or natural products (e.g., chitosan, curcumin, henna) [24]. Although very rare, there are also reports on immobilization of bacteriolytic enzymes, like lysozyme or lysostaphin, on different wound dressings, including nanofibers [25,26,27,28].

Immobilization of bioactive compounds, like enzymes, is very challenging. Special attention must be paid as peptides and proteins are very fragile molecules that need proper three-dimensional structures to sustain their activity. Adsorption and layer-by-layer assembly are based on the physical binding of peptides through noncovalent interactions between fibers components and peptides. Therefore, they do not provide sufficient control on the desorption kinetics of the immobilized molecules [29]. This limitation can be overcome by the covalent binding of bioactive molecules, providing long-term stability of the functionalized materials [30]. The covalent binding may be achieved by using two main strategies: “graft to” and “surface-initiated” methods [23,29]. The first one is based on surface activation by, e.g., UV, plasma treatment, oxidation, etc., while the latter relies on polymerization of monomers bonded to surface initiators. Chemicals used in covalent bonding formation can influence fibers structure and properties, but first of all, they might impede the bioactive molecules. The fact that there are very few reports on the successful immobilization of proteins, particularly enzymes, on the fibers’ surfaces illustrates how challenging this process is [31,32,33].

The aim of this research was to generate the nonwovens functionalized with Auresine*Plus* displaying potent antimicrobial activity. We have compared three methods for molecule immobilization and evaluated their effects on antibacterial efficacy, physical properties, and cytotoxicity. As a result, we have generated nanomaterials functionalized with enzybiotic that display very high efficiency in eradicating *S. aureus* and can serve as the wound dressings of the new generation.

## 2. Materials and Methods

### 2.1. Reagents

For nonwoven preparation poly(lactide-*co*-glycolide) (PLGA) (M_w_ = 190,000–240,000, Resomer 855S, Evonik, Essen, Germany) in blend with chitosan (Heppe Medical Chitosan, Halle, Germany) (low molecular weight, DDA = 77.6–82.5%, 80/50) was used in weight ratio 95:5. As a solvent 1,1,1,3,3,3-hexafluoro-2-propanol (HFIP) (Iris Biotech GmBH, Marktredwitz, Germany) was used. For surface modification following reagents were used: Auresine*Plus* (recombinant enzyme produced in *E.coli* system, 15), 0.1 N NaOH solution (BioXtra, ≥98%, Sigma Aldrich, Munich, Germany), 0.5% glutaraldehyde solution (GA) (25% CZ, POCh, Gliwice, Poland), *N*-hydroxysuccinimide (NHS; 98%, Merck, Darmstadt, Germany) and *N*-(3-Dimethylaminopropyl)-*N*′-ethylcarbodiimide hydrochloride (EDC; 99%, Sigma Aldrich, Munich, Germany), MES (MES hydrate, 99% for biochemistry, Pol-aura, Różnowo, Poland). For antibacterial tests and cellular studies following reagents were used: Phosphate buffer saline (PBS) (Gibco^TM^, Thermo Fisher Scientific, Waltham, MA, USA), DMEM (Gibco^TM^, Paisley, UK), Fetal Bovine Serum (FBS) (Gibco^TM^, Grand Island, NE, USA), antibiotic: Penicillin and streptomycin (10,000 U/mL, Gibco^TM^, Grand Island, NE, USA), 2.5% glutaraldehyde solution (25% CZ, POCh, Gliwice, Poland), ethanol (Chempur, Piekary Sląskie, Poland), hexadimetylosiloxans (Sigma Aldrich, Munich, German), paraformaldehyde (Merck, Darmstadt, Germany), ActinGreen™ and NucBlue™ Reagent (Invitrogen^TM^, Thermo Fisher Scientific, Waltham, MA, USA), Live/Dead Viability/Cytotoxicity Kit for Mammalian Cells (Thermo Fisher Scientific, Waltham, MA, USA), PrestoBlue Cell Viability Reagent (Invitrogen^TM^, Thermo Fisher Scientific, Waltham, MA, USA), MicroBCA Protein Assay Kit (Thermo Fisher Scientific, Waltham, MA, USA), trypticase soy broth (TSB, Merck, Darmstadt, Germany), EthyleneDiamineTetraAcetic acid (EDTA, Merck, Darmstadt, Germany).

### 2.2. Electrospinning

Chitosan was dissolved in 98.5% HFIP for 24 h at 50 °C in a water bath. After 24 h, the solution was cooled down to ambient temperature, PLGA was added and left for dissolution for another 24 h in ambient conditions. The total polymer concentration was set as 5% *w*/*w*. Electrospinning was conducted on custom made equipment (IPPT, Warsaw, Poland) with an applied voltage of +18 kV and a flow rate of 1500 µL/h. The needle-collector distance was set at ca. 12 cm. An 18G needle and a rotating drum collector were used. The collector rotation was 200 rpm and did not lead to any preferred orientation of the fibers. The process was conducted at room temperature and humidity of 35 ± 5%. Nonwoven was left for 7 days to allow the residual solvent to evaporate.

### 2.3. AuresinePlus Immobilization

The Auresine*Plus* was produced as described previously [15]. 1 mg/mL solution of the enzyme in PBS was used for immobilization. Nonwoven was sliced into samples and subjected to sterilization by 80% ethanol solution and UV for 30 min. After sterilization, the samples were incubated in water for 1 h for initial wetting. The control sample, prepared as described above, was called PLGA/C. The remaining samples were immersed in 0.1 N NaOH for 5 min, rinsed with deionized water a minimum of 3 times, until neutral pH was reached, and divided into three groups. The first group was immersed in Auresine*Plus* solution (NaOH/Auresine*Plus*). The second group was incubated in NHS/EDC solution (NHS-0.04% *w*/*v*, EDC-0.27% *w*/*v*) and the third group in 0.5% *v*/*v* GA solution for 1 h [34,35]. After this step, samples were rinsed in PBS. Half of the samples from the second and the third group were dried and saved as a control (NE and GA, respectively). The remaining samples were incubated in Auresine*Plus* solution for 24 h at 4 °C and finally washed 3 times for 10 min in PBS. Samples with attached Auresine*Plus* and immersed previously in NHS/EDC were labeled as NE/Auresine*Plus*, and partly immersed in glutaraldehyde were marked as GA/Auresine*Plus*. Overall, each 1 mg of nanomaterial was incubated with 0.14 mg of the enzyme.

### 2.4. Nanomaterial Characterization

#### 2.4.1. Field Emission Scanning Electron Microscope (FE-SEM)

Nova NanoSEM 450, (FEI, Hilsboro, OR, USA) was used for fibers surface imaging. The imaging was operating at an accelerating voltage of 10 kV. Before imaging, the samples were coated with an 8 nm thick gold layer to minimize charging.

#### 2.4.2. Attenuated Total Reflectance Fourier Transform Infrared Spectroscopy (ATR-FTIR)

ATR-FTIR was performed using Vertex70 spectrophotometer (Bruker, Billerica, MA, USA) to reveal differences in the chemical composition of analyzed samples. Thirty-two scans in the range of 400–4000 cm^−1^ were collected with a resolution of 2 cm^−1^ for all samples. The diamond crystal was used in the spectrometer setup.

#### 2.4.3. Protein Determination

The protein attachment efficiency was analyzed using the microBCA™ Protein Assay Kit (23235, Thermo Fisher Scientific, Waltham, MA, USA). To determine the amount of Auresine*Plus* attached to the fibers surface, samples were placed in 1 mL of microBCA working reagent and incubated for 2 h at 37 °C. Then 225 µL of the solution was placed in a 96-well plate, and the absorbance was measured at 562 nm using Microscan Go UV–Vis spectrophotometer (Thermo Fisher Scientific, Waltham, MA, USA). A purple-colored reaction product is formed by the chelation of two molecules of reagent (BCA) with one cuprous ion (Cu^+1^) reduced by protein in alkaline conditions. The same procedure was used to prepare the calibration curve for solutions with known protein concentrations.

#### 2.4.4. Differential Scanning Calorimetry (DSC)

DSC analysis was performed on the Perkin Elmer PYRIS-1 device (Perkin Elmer, Waltham, MA, USA). The DSC was performed to verify changes in the supramolecular structure of nonwovens caused by various methods of Auresine*Plus* immobilization. The measurements start with an isothermal stop at 20 °C per 5 min, followed by heating up to 200 °C at the rate of 10 °C/min. The average sample weight was ca. 2.5 mg. The Origin 8 software (OriginLab Corporation, Northampton, MA, USA) was used for data analysis.

#### 2.4.5. Mechanical Properties

Static tensile tests were conducted to check the influence of the Auresine*Plus* immobilization method on nonwoven mechanical properties. The tests were performed on a Lloyd EZ50 device (Ametek Inc., Berwyn, IL, USA) equipped with grips for small loads. The dry samples used were 2 cm long (l_o_), 0.5 cm wide, and ca. 80 µm thick. The preliminary load was set as 0.2 N, and the sample’s stretching rate 5 mm/min. The measurements were carried out at room temperature and humidity around 40%. Young’s modulus was calculated.

#### 2.4.6. Fiber Surface Properties

The contact angle (CA) and surface energy (SE) were measured using OCA15 Goniometer (Data Physics) (Data Physics Instruments GmbH, Filderstadt, GermanyAll measurements were recorded at room temperature on materials previously dried in a vacuum dryer. A water drop of 1 µL was transferred onto the material, and the contact angle was measured at the moment of the first contact of the drop with the material’s surface. For SE calculation, the measurements were also done using formamide. The SE was calculated using the OWRK theory.

#### 2.4.7. Water Absorption

Water absorption test was done at selected time points for samples dried in a vacuum dryer overnight. After that, samples were incubated in distilled water at room temperature for 15 min, 30 min, and 1, 2, 3, and 12 h. At each time point, samples were slightly dried on a paper towel and weighed. The water absorption was calculated as % of the initial weight of the dry samples.

### 2.5. Cytocompatibility Test

Cytotoxicity of wound dressing modified with Auresine*Plus* was assayed by monitoring mice fibroblast (L929, Sigma-Aldrich, Munich, Germany) viability. Cells were cultured in a medium composed of 90% DMEM, 10% FBS, 1% antibiotic (penicillin, streptomycin). Materials were sterilized using UV radiation for 15 min, and cells were cultured on nonwovens for up to 10 days (37°C, 5% CO_2_). After 1, 3, 7, and 10 days cells’ viability was analyzed using Presto Blue assay (Invitrogen^TM^, Thermo Fisher Scientific, Waltham, MA, USA). The dye was added to each sample in a volume ratio of 1:9 and incubated for 1 h. The data were collected using Fluoroskan Ascent FL (Thermo Fisher Scientific), with excitation at 530 nm and emission at 620 nm.

### 2.6. Adhesion and Proliferation Test

Cell adhesion and morphology were imaged using a scanning electron microscope (JEOL JSM-6390LV, Jeol, Akishima, Tokio, Japan), after crosslinking in 2.5% GA, dehydration with ethanol and ethanol/hexadimetylosiloxan solutions. For fluorescent imaging of actin cytoskeleton and nucleus, samples were fixed with 3% PFA solution for 30 min and then stained with Alexa Fluor 488 dye (ActinGreen™, Invitrogen^TM^) and Hoechst 33342 (NucBlue™ Reagent, Invitrogen^TM^). The observations were carried out using a fluorescent microscope (Leica DMI3000B, Leica Microsystems, Wetzlar, Germany). The live/dead cell imaging was done using LIVE/DEAD™ Viability/Cytotoxicity Kit (Invitrogen^TM^). After 1 and 3 days of cell culture, the samples were rinsed with PBS and incubated for 15 min in calcein and ethidium homodimer-1 solution. Finally, samples were again rinsed with PBS and imaged using a fluorescent microscope (Leica DMI3000B, Leica Microsystems). The calcein interacts with live cells (stained green), and ethidium homodimer-1 interacts with dead cells (stained red).

### 2.7. Bacterial Cultures

The single colony of *S. aureus* NCTC 8325-4 was inoculated in small-volume overnight culture in TSB medium and cultivated at 37 °C with 80 rpm agitation. The next day, the fresh portion of medium was inoculated with 2% of the night culture and grown until the culture reach the exponential phase of growth (OD_600_ around 0.6, ~10^8^ colony-forming units per mL, CFU/mL). Bacteria were collected by centrifugation, and the pellet was resuspended to around 1 × 10^6^ CFU/mL in PBS. The enzyme lytic activity released or covalently bound to the wound dressings was assessed based on the plating assays. The procedure was adapted from Verbree et al. [36].

### 2.8. Contact Assay

Each nanomaterial variant was cut into small pieces of approx. 0.5 cm^2^, and each of them was placed separately into the bottom of an empty mini-column routinely used for DNA extraction. The samples were duplicated and further treated as technical repetition for each nanomaterial. 50 µL of bacteria suspension in PBS was placed directly on each nanomaterial sample and incubated for 3 h at room temperature. The bacterial suspension was physically separated from nanomaterials by centrifugation for 1 min at 3.5 k rpm, and the flow-through was collected for bacteria number determination. In order to inactivate the enzyme that was released, EDTA, the inhibitor of the metallopeptidases, was added to each fraction to the final concentration of 1 mM. The number of bacterial cells in the samples was evaluated by plating 10-fold serial dilutions in PBS. For preliminary estimation of the bacterial cell number, 5 µL of each dilution was spotted in rows on TSB-A plates (Greiner Bio-One GmbH, Rainbach im Mühlkreis, Austria). For calculation of the exact number of bacterial cells, the 100 µL of each dilution was evenly spread on TSB-A plates. After overnight incubation at 37 °C, the number of bacteria expressed as CFU/mL was calculated and compared to the control sample of bacterial suspension not subjected to the wound dressing treatment. The enzymatic activity was calculated as differences between them.

### 2.9. Release Assay

To release the protein from electrospun materials, they were cut into 1 cm^2^ pieces and subjected to the serial washing steps with 200 µL filtered PBS. Samples were placed on rotation in the programmable Rotator Multi Bio RS-24 (BioSan, Riga, Latvia) at 4 °C to provide the optimal conditions for protein stability and exclude the risk of its degradation at late time points. At selected time points, 200 µL of the supernatant was collected, and replaced with an equal fresh portion of the PBS. The experiment was performed in a technical triplicate. The antibacterial activity was determined by mixing the released sample and the bacteria suspension in PBS in 1:1 (*v*/*v*) proportion for 3 h at room temperature. The reaction was stopped by adding 1 mM EDTA to inactivate the enzyme, and the sample was subjected to bacterial number determination as described above.

### 2.10. Enzyme Content Estimation

Since the protein concentration in samples released from the nanomaterials verified on Nanodrop gave very low protein concentration values, we seek to estimate it using a calibration curve prepared based on the activity of Auresine*Plus* in solution (Figure S1), that enabled us to calculate the total amount of the released, enzymatically active protein. To exclude the possible antibacterial effect of the nanomaterial alone, the same procedure was implemented for the negative controls (nanomaterial without enzyme adsorbed) and subtracted from the results obtained for samples functionalized with the enzyme.

### 2.11. Statistical Analysis

The quantitative data were expressed as mean ± SD and were compared using a one-way ANOVA test with analysis of variance (Tukey’s test). The data were considered significantly different when *p* < 0.05. For direct contact antimicrobial assay, the average and standard deviation were calculated based on two independent assays, each performed using three nanomaterial samples. The release assay was performed once on three nanomaterial samples.

## 3. Results and Discussion

**Figure 1:**
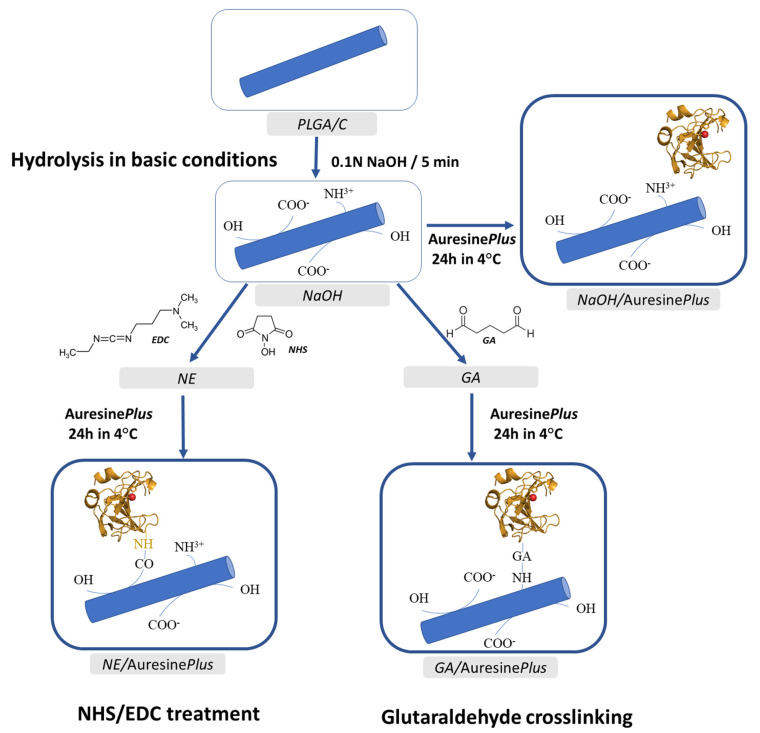
Scheme of the electrospun fiber surface modification and the enzyme attachment procedures used in this study. The details of the procedures are described in Section 2.

Proteins, particularly enzymes, are very fragile macromolecules. They have to keep the integrity and proper structure to sustain their activity. Hence, the immobilization of enzymes on fibers that require chemical modification is a real challenge. Although there are many possible fiber functionalization methods, only very few were reported to be successfully used for protein immobilization [26,27,28]. We have tested three different immobilization methods to find the most effective and at the same time safe for the enzyme: physical adsorption on the previously hydrolyzed surface and covalent bonding using *N*-hydroxysuccinimide/*N*-(3-Dimethylaminopropyl)-*N*′-ethylcarbodiimide solution (NHS/EDC) or glutaraldehyde solution (GA) (Figure 1).

### 3.1. Fibers Morphology

The average diameter for PLGA/chitosan fibers was calculated as 517 ± 29 nm. Pure PLGA fibers prepared using the same processing parameters would exhibit a much higher average diameter, ca. 1.5 µm [23]. Thanks to chitosan addition, the fibers average diameter and pore size is smaller—making the nonwoven more similar to the extracellular matrix and creating a better mechanical barrier for the microbes and other wound contaminations. Additionally, chitosan provides much better nonwovens wettability, and by that proper hydration of the wound, tissue stimulation for healing (wound closure), and neutralization of pH, if it becomes too acidic [37].

**Figure 2:**
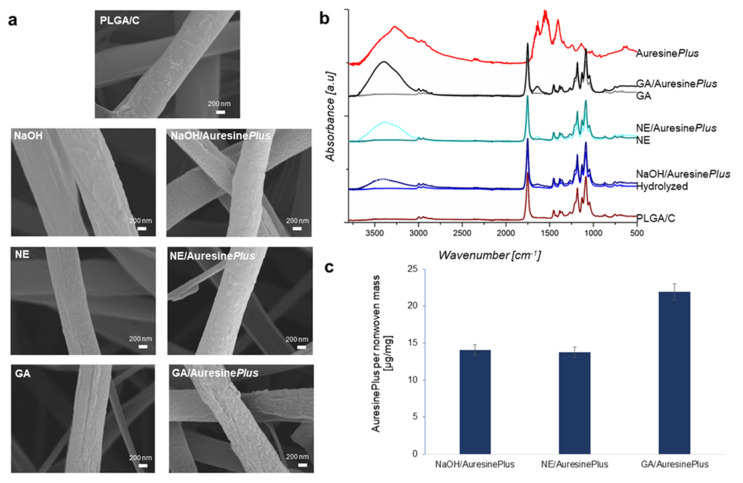
Analysis of Auresine*Plus*-functionalized nonwovens. (**a**) Field emission scanning electron microscope (FE-SEM) images illustrating the surface of bare fibers (PLGA/C), as well as fibers subjected to various methods of Auresine*Plus* immobilization. (**b**) Spectra obtained by infrared spectroscopy (ATR-FTIR), presenting differences in the chemical composition of analyzed samples. (**c**) Quantitative estimation of Auresine*Plus* immobilized on the nonwovens using microBCA Protein Assay Kit.

**Figure 3:**
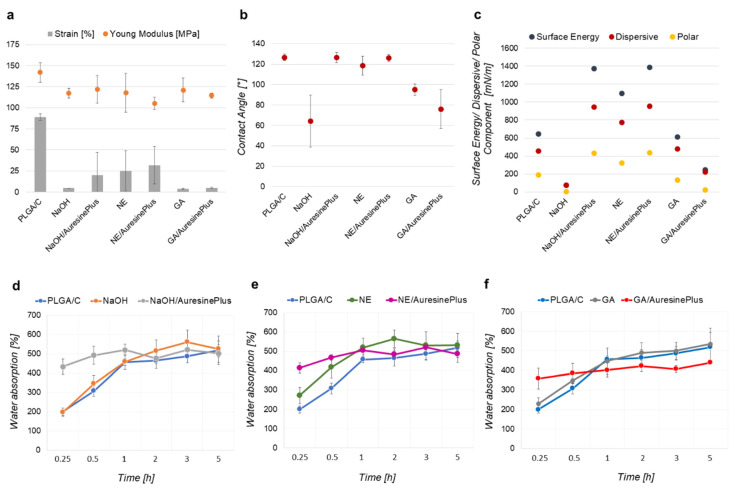
Mechanical properties and surface properties analysis. (**a**) Static tensile test conducted for nonwovens. (**b**) Contact angle measured with water drop at ambient conditions. (**c**) Surface free energy calculated usIOWRK theory. (**d**–**f**) Water absorption of nonwovens both, control and samples with immobilized Auresine*Plus.* (**d**) Hydrolyzed in basic conditions. (**e**) Treated with NHS/EDC. (**f**) Treated with glutaraldehyde.

High-resolution imaging by FE-SEM revealed differences between PLGA/C and all materials subjected to Auresine*Plus* immobilization (Figure 2a, Figure S2). The surface of the control sample (PLGA/C) was relatively uniform with locally visible fractures. After hydrolysis in basic conditions, the surface lost uniformity indicating degradation and activation of the fibers. This observation was also supported by DSC data (Table 1), proving a strong effect of the treatment on the supramolecular structure of PLGA, reflected in characteristic temperatures and phase transition changes. These changes negatively influence the mechanical properties of the nonwovens, decreasing their elasticity (Figure 3). Degradation of PLGA and increase of the surface roughness as an effect of hydrolysis in basic conditions were previously reported for PLGA films [38]. In nanofibers, the damage to the surface can be even more pronounced, as the processing conditions could also affect the structure across the fibers. On the other hand, a more developed surface topography of the fibers can be beneficial for effective Auresine*Plus* immobilization (Figure 2). After incubation in Auresine*Plus* solution, a visible, new layer appeared on each fibers sample surface (Figure 2a).

### 3.2. Chemical Composition

The chemical composition of the nonwovens was analyzed by ATR-FTIR (Figure 2b) and microBCA assay (Figure 2c). The infrared spectra of Auresine*Plus* exhibited several characteristic peaks: In the range, 1700–1600 cm^−1^ assigned to amide I and 1600–1500 cm^−1^ assigned to vibration in N–H bond in amide II. The displayed maximum of amide I at 1638 cm^−1^ is predominantly assigned to the β-sheet structured protein. A peak at 1551 cm^−1^ indicates the high content of tyrosine residues. Other peaks are also present on Auresine*Plus* spectra, e.g., broad peak in the range 3600–2400 cm^−1^ within which vibrations from following bonds are present: N–H bond, stretching vibrations of the O–H, both from protein and absorbed water, the absorption spectra at 2962 cm^−1^ assigned to the asymmetrical C–H stretching vibrations of aliphatic CH_2_ and the spectra at 1435 cm^−1^ to the C–H bending of aliphatic CH_2_. The peaks in the range 1500–900 cm^−1^ come from the CH_3_, O–H deformational vibrations, and C–O–C bond.

For PLGA/C, the most characteristic peaks in the fingerprint region are from PLGA component—1752 cm^−1^ assigned to C=O, both in lactide and glycolide component, and 1423 cm^−1^ corresponding to C–H deformation in the –O–CH_2_ bond of the glycolide component [39]. The chitosan peaks are not visible on the spectra, due to the low content of chitosan in the PLGA/C sample.

Samples NaOH/Auresine*Plus*, NE/Auresine*Plus*, and GA/Auresine*Plus* revealed a clear peak in the range of 1700–1500 cm^−1^ (amide I and amide II), confirming the presence of immobilized Auresine*Plus* on these samples. Moreover, additional peaks are observed in the range 3600–3000 cm^−1^ that are correlated with significant absorbance of water (Figure 2b).

The microBCA assay was performed to estimate the Auresine*Plus* amount attached to PLGA/C nonwovens (Figure 2c, Figure S3). This analysis showed that around 14 µg of Auresine*Plus* was present in 1 mg of NaOH/Auresine*Plus* and NE/Auresine*Plus* samples. On GA/Auresine*Plus*, the highest amount of the enzyme detected was approx. 22 µg/mg. These two covalent protein immobilization methods were also used by Miao et al. [28] for functionalization of cellulose fibers with lysostaphin, giving similar efficiencies of enzyme attachment.

### 3.3. Immobilization Methods

**Figure 4:**
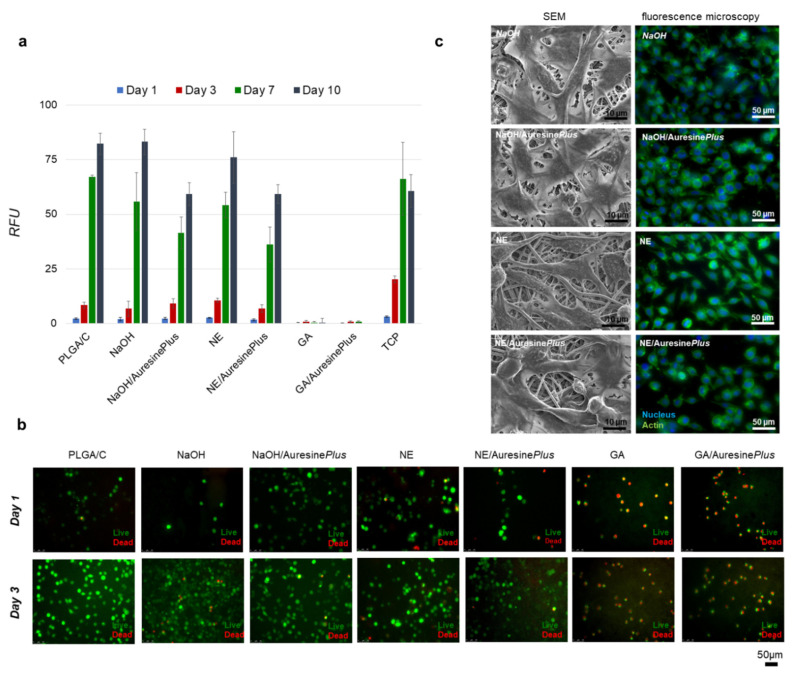
Cytotoxicity analysis of functionalized nanomaterials. (**a**) Fibroblasts viability analyzed up to 10 days of cell culture using Presto Blue assay for all types of samples, TCP—tissue culture plastic, RFU—relative fluorescence units. (**b**) Visualization of the fibroblasts viability during the first 3 days of growth on nonwovens. Live cells (green), dead cells (red). (**c**) Morphology of fibroblasts grown on nonwovens samples visualized by SEM and fluorescent microscopy; actin cytoskeleton (green) and nucleus (blue). Images were collected after 3 days of culture.

**Figure 5:**
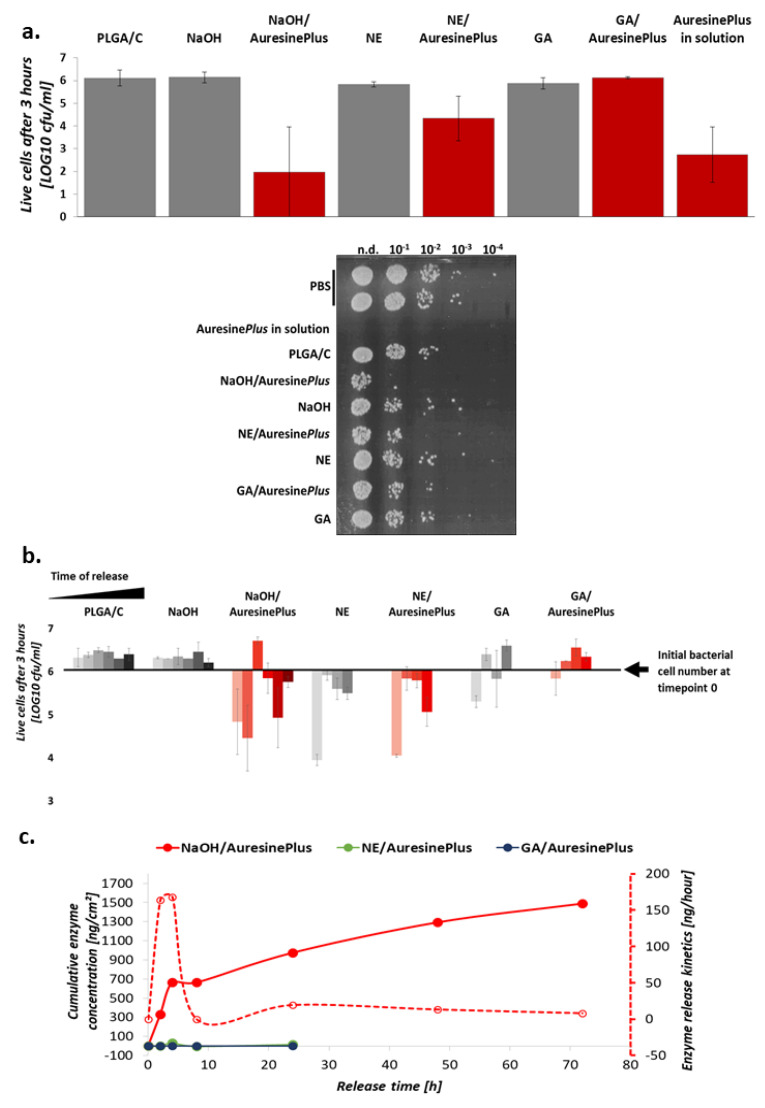
Antimicrobial activity of functionalized materials. (**a**) *S. aureus* NCTC 8325-4 viability upon direct-contact with nonwovens with immobilized Auresine*Plus* and respective controls. Around 10^6^ CFU/mL of bacterial suspension in PBS was incubated on the nonwoven surface for 3 h to observe the most prominent differences between studied variants. Antimicrobial activity documentation image shows the viability of bacteria at direct-contact assay. (**b**) Antibacterial activity of the enzyme upon release from nanomaterials. The lytic effect was observed upon release in a time course. The release was conducted for all samples at 2, 4, 8, and 24 h; for NaOH, NaOH/Auresine*Plus*, and PLGA/C, it was extended up to 72 h to better study the kinetics of the protein release from this variant (physical adsorbtion), and due to prominent antibacterial activity of the enzyme in NaOH/Auresine*Plus* at 24 h time point in contrast to NE/Auresine*Plus* and GA/Auresine*Plus*. The conditions of the reaction were the same as in a. For (**a**,**b**), the values were log transformed, averaged and the SD was calculated based on transformed data. (**c**) Release of the enzyme from the nonwovens. The amount of the enzyme in the samples collected at the indicated timepoint was estimated based on the observed activity (see Section 2). In the dotted line, the kinetics of the enzyme release is indicated and presented as an amount of enzyme release in time periods estimated based on the antimicrobial activity of released fraction at each time point.

Physical immobilization is the least invasive method, which is particularly important in loading nanomaterials with bioactive compounds, like enzymes [40,41]. Simultaneously, this method was reported to have a limitation when it comes to controlling the active molecule release by desorption [16]. In our case, hydrolysis had visibly expanded nanomaterial surface compared to control PLGA/C fibers, and therefore, could accommodate substantial amounts of the enzyme (Figure 2a–c). Two other methods used in our experiments (NHS/EDC and GA) resulted in the formation of the covalent bonding between the enzyme and the fiber surface. NHS/EDC treatment had comparable efficiency in Auresine*Plus* immobilization as physical adsorption. However, GA and GA/Auresine*Plus* became cytotoxic (Figure 4a). Moreover, as it will be proved later (Figure 5c), the release of enzymes is not observed. Despite the highest amount of enzyme immobilization by GA treatment, this procedure significantly lowered the antibacterial activity of Auresine*Plus* (Figure 5a–c).

Summarizing, the ATR-FTIR spectra confirmed immobilization of Auresine*Plus* on all functionalized fibers; strong amide I and amide II peaks, characteristic for Auresine*Plus*, were visible in the range of 1700–1500 cm^−1^. Although the highest enzybiotic amount was immobilized using the GA method (Figure 2c), their antimicrobial activity was almost completely diminished. Moreover, these functionalized fibers displayed potent cytotoxic effects, probably due to the remaining GA (Figure 4a). Similar adverse effects have been reported [30], but there are also cases of successful application of this method for enzyme immobilization [28].

### 3.4. Characterization of Nonwoven Structure and Mechanical Properties

Differential scanning calorimetry (DSC) was performed to detect possible changes in the supramolecular structure of PLGA/C caused by enzyme immobilization procedures. The glass transition temperature (T_g_) for PLGA/C was determined as 41.1 °C. The T_g_ of all nonwovens after enzyme immobilization increased significantly to 46–48 °C (Table 1). There are no significant heat capacity changes (ΔCp) during glass transition observed, due to the immobilization process or protein presence. Due to the T_g_ shift, there is also a clear shift of relaxation temperature toward higher values for all samples comparing to PLGA/C (Table 1). Significant changes also occurred in the PLGA crystal phase. The T_m_ of all analyzed nonwovens increased compared to PLGA/C, on average by 1.2 °C. The simultaneous growth of T_g_ and T_m_ indicates polymer chain backbone stiffening. The analysis of heat of fusion changes also indicates the significant influence of chemical treatment on PLGA crystallinity. The hydrolysis in basic conditions caused a significant increase of heat of fusion for cold crystallization of PLGA in NaOH and NaOH/Auresine*Plus* samples compared to PLGA/C. This observation indicates increased polymer chain mobility arising from polymer chain scission during NaOH treatment. Later on, during NHS/EDC and GA treatment, PLGA chains were crosslinked. For this reason, the heat of fusion for cold crystallization is significantly lower for all glutaraldehyde and NHS/EDC treated samples than for nonwovens subjected only to hydrolysis in basic conditions (Table 1).

Consequently, these materials become more rigid than control (Figure 3a), and as such, may still find applicability as a wound dressing. The DSC analysis proved significant glass transition changes (T_g_) and the heat of fusion (ΔH_Tk_ and ΔH_Tm_) values for each nonwoven, indicating that processing conditions influence PLGA chains length, mobility, and PLGA crystallinity. These changes might be significant for designing storage conditions and the applicability of the nonwovens.

Figure 3a illustrates data obtained in the static tensile test for analyzed nonwovens. The Young modulus decreased about 20–37 MPa for all samples compared to the control PLGA/C, which is 85–75% of the initial value. At the same time, the strain decreased by approx. 65–95%. The average maximal stress, which may be applied to each sample, is 3.5 Mpa. These data confirm significant polymer structure changes caused by chemical treatment during enzyme immobilization and prove its direct correlation with nonwoven functional properties (Table 1).

One of the essential features of wound dressings are the surface properties. To characterize the surface properties of analyzed nonwovens, each sample’s contact angles were measured (Figure 3b). The hydrolysis in sodium hydroxide caused the most significant decrease in the contact angle by 65°, making this sample very hydrophilic. This effect is most probably connected with PLGA chain scission during hydrolysis in basic conditions (Table 1). The wettability of the NaOH sample was in the range that is optimal for the highest cell adhesion [42].

Interestingly, incubation in Auresine*Plus* solution increased the contact angle to ca. 126° (NaOH/Auresine*Plus*), the value corresponding to the one measured for the control sample (PLGA/C). Treatment with glutaraldehyde increased contact angle to 95°, while NHS/EDC up to 126°, combined with PLGA crosslinking in fibers bulk (Table 1). The immobilization of enzybiotic on NaOH/Auresine*Plus* and NE/Auresine*Plus* samples did not cause an increase in its hydrophilic character. Only GA/Auresine*Plus* sample becomes hydrophilic after immobilization (Figure 3b). It is worth noticing that CA was measured during a water drop with a nonwoven surface during the first contact. CA data correlates with SE, and nonwovens with Auresine*Plus* seem to be hydrophobic at the moment of data collection. However, as water absorption curves show (Figure 3d–f), the water infiltrates the nonwovens in the time course. In the case of PLGA/C, the drop stayed on nonwovens surface for a very long time, until complete evaporation, while NaOH/Auresine*Plus*, NE/Auresine*Plus*, and NE absorbed the water within the first 10 s.

Auresine*Plus* immobilization caused an increase of surface energy and consequently an increase of the contact angle. The contact angle and surface free energy values of NE/Auresine*Plus* are also higher than NE, indicating effective Auresine*Plus* immobilization (Figure 3b,c). It is worth noticing that despite a similar contact angle, these nonwovens cannot be considered as hydrophobic as PLGA/C. The differences in these materials’ behavior in contact with water drop are much different, due to differences in the surface free energy value and its components (polar, dispersive) (Figure 3b,c).

Each method of surface activation had a different effect on its surface free energy. NaOH treatment drastically decreased the surface free energy that might be assigned to significant PLGA degradation and increase of fibers surface roughness; the NHS/EDC and GA treatment resulted in its increase comparing to this value for NaOH sample, probably due to crosslinking of PLGA molecules [43]. The Auresine*Plus* immobilization increased the total surface energy of NaOH/Auresine*Plus* and NE/Auresine*Plus* (Figure 3c), but not in GA/Auresine*Plus* samples, which might result from differences in the amount of attached enzybiotic and/or influence of this procedure on Auresine*Plus* conformation and activity (Figure 2).

Water absorption is an essential feature of wound dressings. In all control samples (without enzyme), the water absorption was low initially and reached a maximum during the experiment’s first hour. In the case of samples with immobilized Auresine*Plus*, the water absorption was much higher at the beginning, compared to the controls. However, by the end of the experiment, all samples reached similar water saturation. Additionally, ATR-FTIR spectra confirmed water absorption (vibrations from O–H bond in the range 3500–3000 cm^−1^) by samples with Auresine*Plus*, which may be beneficial for wound hydration during the healing process (Figure 3).

### 3.5. Cytocompatibility, Adhesion and Proliferation Test

To verify the nonwoven biocompatibility with immobilized Auresine*Plus*, the fibroblast viability cultured on these materials was analyzed using Presto Blue assay (Figure 4a). Only in the case of GA and GA/Auresine*Plus* samples, an apparent cytotoxic effect was observed already on the first day of the experiment. During ten days of the experiment, no cells were detected on these nonwovens. As this effect was observed in both control and samples with immobilized enzymes, the cytotoxicity effects are more likely to result from the functionalization procedure itself. The other materials exhibited biocompatibility; however, the cell proliferation on these nonwovens is slower than on Tissue Culture Plastic (TCP). This might be explained by the different topography and mechanical properties of nonwoven compared to polystyrene plates. During ten days of the experiment, fibroblasts constantly proliferated similarly on all tested samples. However, fewer cells were detected on NaOH/Auresine*Plus* and NE/Auresine*Plus* than on the corresponding controls (Figure 4a).

The cytotoxicity of the nonwovens was also evaluated by fluorescence microscopy. Live cells stained with calcein (green) dominated images of all samples except GA and GA/Auresine*Plus* (Figure 4b). Most of the cells detected on these two materials were stained by ethidium homodimer-1 as dead (red). On the rest of the samples, both controls, and immobilized Auresine*Plus*, most of the cells were alive, and only single dead cells were found. Additionally, more cells were classified as alive after three days comparing to day 1, indicating continuous proliferation of fibroblasts on the nonwovens (Figure 4b).

The fibroblasts morphology was examined after 3 and 7 days of cell culture on nonwovens and compared to cells grown on TCP (Figure 4c, Figure S4). Only on GA and GA/Auresine*Plus* materials cells were not present. Fibroblast had a well-developed actin cytoskeleton (green) and a well visible, centrally located nucleus (blue). Various shapes of cells illustrating different stages of development were observed. Mature, spindle-like, and well-flattened cells were present, as well as young, still round ones (Figure 4c).

The biocompatibility of the wound dressings is one of the most crucial features. Our tests showed clearly that only the GA and GA/Auresine*Plus* fibers were cytotoxic. This effect was observed probably due to glutaraldehyde residues still present on the nonwovens. Similar adverse effects have been reported previously [30], but successful application of this method for enzyme immobilization was also observed [28].

### 3.6. Antimicrobial Activity

To test which of the immobilization methods is the best in respect of sustaining antimicrobial activity of the enzyme, the lytic activity of the generated materials was evaluated. Direct antibacterial effects of the materials were tested in contact assays, and it showed that materials with physically adsorbed enzyme on previously hydrolyzed fibers surface (NaOH/Auresine*Plus*) displayed the highest bacteriolytic activity. The efficiency in eradicating staphylococcal cells was at the same level as 25 nM Auresine*Plus* in solution (Figure 5a). It was calculated that 9 ± 4 × 10^5^ (99.4 ± 0,6%) bacteria were eliminated from 0.25 cm^2^ of the material surface in 3 h (Figure 5a, Table S1). The NE/Auresine*Plus* nonwoven with covalently attached enzyme also displayed potent antistaphylococcal activity. No antibacterial effects were observed on GA/Auresine*Plus* samples. The effects of the control materials without enzyme were negligible; hence, the antimicrobial effects demonstrated in the contact assays are attributed to the immobilized enzyme activity. Two independent assays gave similar results indicating high reproducibility of nonwovens preparation and their functionalization (Table S1).

The enzyme was immobilized on nanomaterials either physically (NaOH/Auresine*Plus*) or covalently (NE/Auresine*Plus*, GE/Auresine*Plus*), and the way of enzyme attachment was expected to influence the kinetics of its release. Therefore, we measured the release of Auresine*Plus* from each nonwoven variant over time by estimating the amount of released protein based on the bacteriolytic activities exhibited by NaOH/Auresine*Plus* (Figure 5b,c).

As expected, the most significant amounts of the enzyme are released from NaOH/Auresine*Plus*, and the release is the most rapid during the first 4 h and then slows down (Figure 5c). Although most of the enzymes are released early, we still observed the main antibacterial activity in fractions collected after 72 h, meaning that this nanomaterial variant keeps its lytic proprieties for an extended time.

Negligible amounts of enzyme were released from covalently bound materials (NE/Auresine*Plus*, GA/Auresine*Plus*) (Figure 5c). The sample collected in the first two hours of release assay from both NE and NE/Auresine*Plus* materials that displayed relatively high antibacterial effects (Figure 5b) could not be attributed to the released enzyme activity and very likely derived from other soluble compounds used in the process of functionalization. To summarize, presented data show that the Auresine*Plus* immobilized either by covalent binding or physical adsorption on PLGA/C nanofibers display potent antibacterial activity.

The fabricated electrospun nonwovens present another example of a successful approach combining the potent activity of enzybiotics with the biomaterials suitable for designing wound dressings [5]. Both antibacterial assays, the direct-contact assay and the one performed on released fractions, gave very consistent results, indicating the most potent antimicrobial effects of NaOH/Auresine*Plus* fibers, which were able to eliminate about 10^6^ bacterial cells in 3 h (Table S1). This material antimicrobial effectiveness was comparable to the activity of 25 nM Auresine*Plus* in solution and is at a similar level as cellulose fibers functionalized with lysostaphin, another bacteriolytic enzyme with the same specificity [28]. This result can be easily correlated with the amount of enzyme released (Figure 5c).

Moreover, we have observed a somewhat limited, but constant release of Auresine*Plus* from nonwovens with an initial burst of release occurring between 4 and 8 h. The most significant amount of enzyme was released in the first 4 h, and then the process slowed down. Such kinetics corresponds very well to the needs of infected wound treatment when the boost of antibacterial activity is needed at the beginning of the treatment to eliminate the infection. Later, the lower concentration of antibacterial agents is sufficient to prevent bacterial contamination.

As compared to NaOH/Auresine*Plus* variant, NE/Auresine*Plus* exhibits lower antibacterial activity. This can be explained by the fact that the enzyme is not released from this sample, due to covalent bonding. In contrast, the contacts between the immobilized enzyme and the bacterial cells are limited compared to the solution. Still, enzyme immobilization on the surface of fabricated material is an advantage in the context of safety (diminishing potential cytotoxic effect *in vivo)* or efficiency reasons (limiting the high enzyme action only to the infection site). The extended time of the release of Auresine*Plus* from NaOH in vivo fibers is an additional advantage in bacterial eradication in the wounds.

Even though the protein load was detected in the GA/Auresine*Plus* (Figure 2c), this approach resulted in either enzyme denaturation or the inability of covalent link formation, eventually resulting in a lack of lytic activity. Thus, this particular approach proved not to be suitable for immobilization of Auresine*Plus* to electrospun nonwovens.

## 4. Conclusions

After testing three different immobilization methods of novel enzybiotic, Auresine*Plus*, to the surface of nonwovens prepared via electrospinning, we have generated functionalized antibacterial fibers. We observed that all methods caused significant changes in the structure and properties of poly(lactide-*co*-glycolide)/chitosan fibers and resulted in efficient enzyme immobilization. Auresine*Plus* maintain its potent antibacterial properties when physically attached to the fibers or covalently bonded using the NHS/EDC method. We have demonstrated that the antimicrobial activity of enzybiotic can be successfully combined with electrospun fibers to generated new materials with unique features that can be used for the treatment of wound infections.

## Figures and Tables

**Table 1:** Results of differential scanning calorimetry (DSC) analysis. Abbreviations: T_g_—glass transition temperature; ΔC_p_—heat capacity change during glass transition; T_rel_—relaxation temperature; T_k_—cold crystallization temperature; ΔH_Tk_—the heat of fusion for cold crystallization; T_m_—melting temperature; ΔH_Tm_—the heat of fusion for crystal phase melting.

	T_g_ (°C)	ΔC_p_ (J/gC)	T_rel_ (°C)	T_k_ (°C)	ΔH_Tk_ (J/g)	T_m_ (°C)	ΔH_Tm_ (J/g)
PLGA/C	41.1	0.6	46.5	107.4	30.4	154.1	108.5
NaOH	46.6	0.5	51.9	107.8	99.1	156.4	104.0
NaOH/Auresine*Plus*	45.9	0.5	50.9	106.2	110.9	155.3	113.1
NE	47.9	0.6	52.2	107.6	9.8	155.6	16.8
NE/Auresine*Plus*	46.4	0.5	512	109.0	14.1	155.2	16.0
GA	46.6	0.6	51.5	108.9	13.0	154.6	14.3
GA/Auresine*Plus*	46.4	0.6	51.1	105.3	18.5	154.8	23.2

## Data Availability

Not applicable.

## Supplementary Materials

The following are available online at https://www.mdpi.com/article/10.3390/pharmaceutics13050711/s1, Figure S1: Calibration curve of the Auresine*Plus* antimicrobial activity, Figure S2: Field emission scanning electron microscope (FE-SEM) images illustrating homogeneity of bare fibers (PLGA/C), as well as fibers subjected to various methods for Auresine*Plus* immobilization, Figure S3: Determination of Auresine*Plus* amount attached to 1 mg of nonvowens, Figure S4: Morphology of fibroblast grown on various fibers and control conditions (TCP), Table S1: Antimicrobial activity of the functionalized materials.
