# Supplementary material for: Staphylococcus aureus Specific Electrospun Wound Dressings: Influence of Immobilization Technique on Antibacterial Efficiency of Novel Enzybiotic
Source: Pharmaceutics. 2021 May 13;13(5):711. doi: 10.3390/pharmaceutics13050711 (PMC8152744; doi:10.3390/pharmaceutics13050711)
Supplement: Supplementary file 1 [file pharmaceutics-13-00711-s001.zip › pharmaceutics-1196502-supplementary.pdf]

pharmaceutics

Supplementary Materials: *Staphylococcus aureus* Specific Electrospun Wound Dressings: Influence of Immobilization Technique on Antibacterial Efficiency of Novel Enzybiotic

Olga Urbanek, Alicja Wysocka, Paweł Nakielski, Filippo Pierini, Elżbieta Jagielska and Izabela Sabała

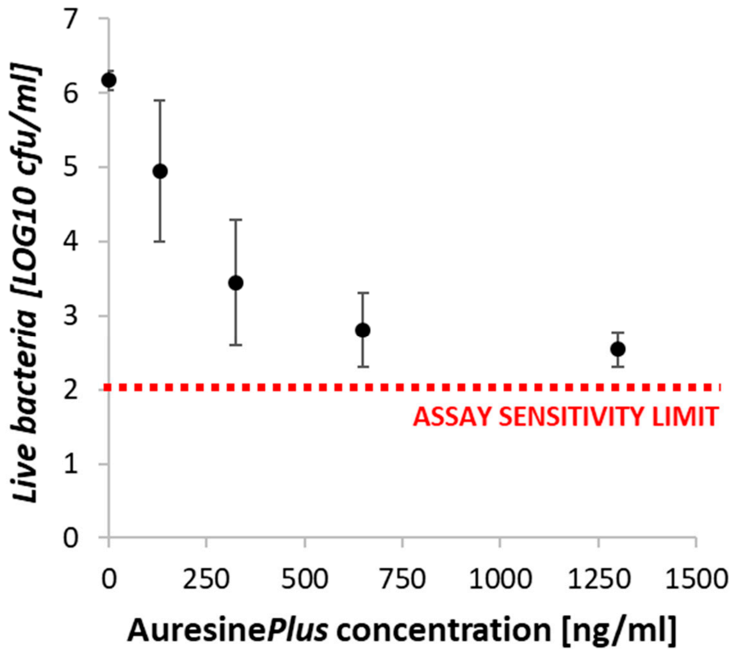

Figure S1. Calibration curve of the Auresine*Plus* antimicrobial activity. *S.aureus* NCTC 8325-4 bacterial cells (10^6 CFU/mL) were subjected to the increasing amounts of the enzyme in the PBS buffer at room-temperature. The reaction was stopped after 3 h by adding the enzyme inhibitor, 1mM EDTA. Presented values show the number of live bacterial cells in log transformed CFU/mL. The bars represent standard deviation from two independent experiments.

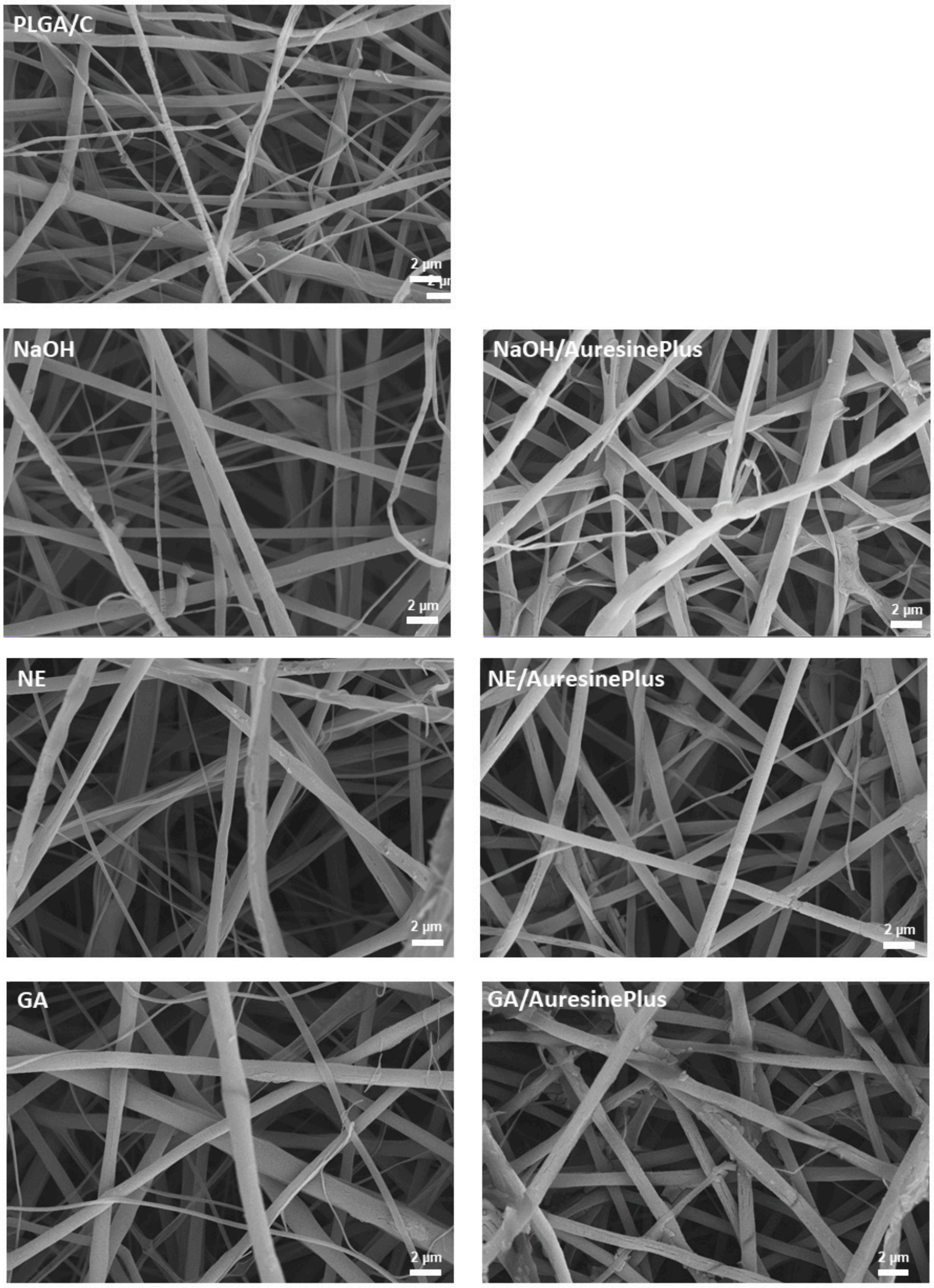

Figure S2. Field emission scanning electron microscope (FE-SEM) images illustrating homogeneity of bare fibers (PLGA/C), as well as fibers subjected to various methods for Auresine*Plus* immobilization.

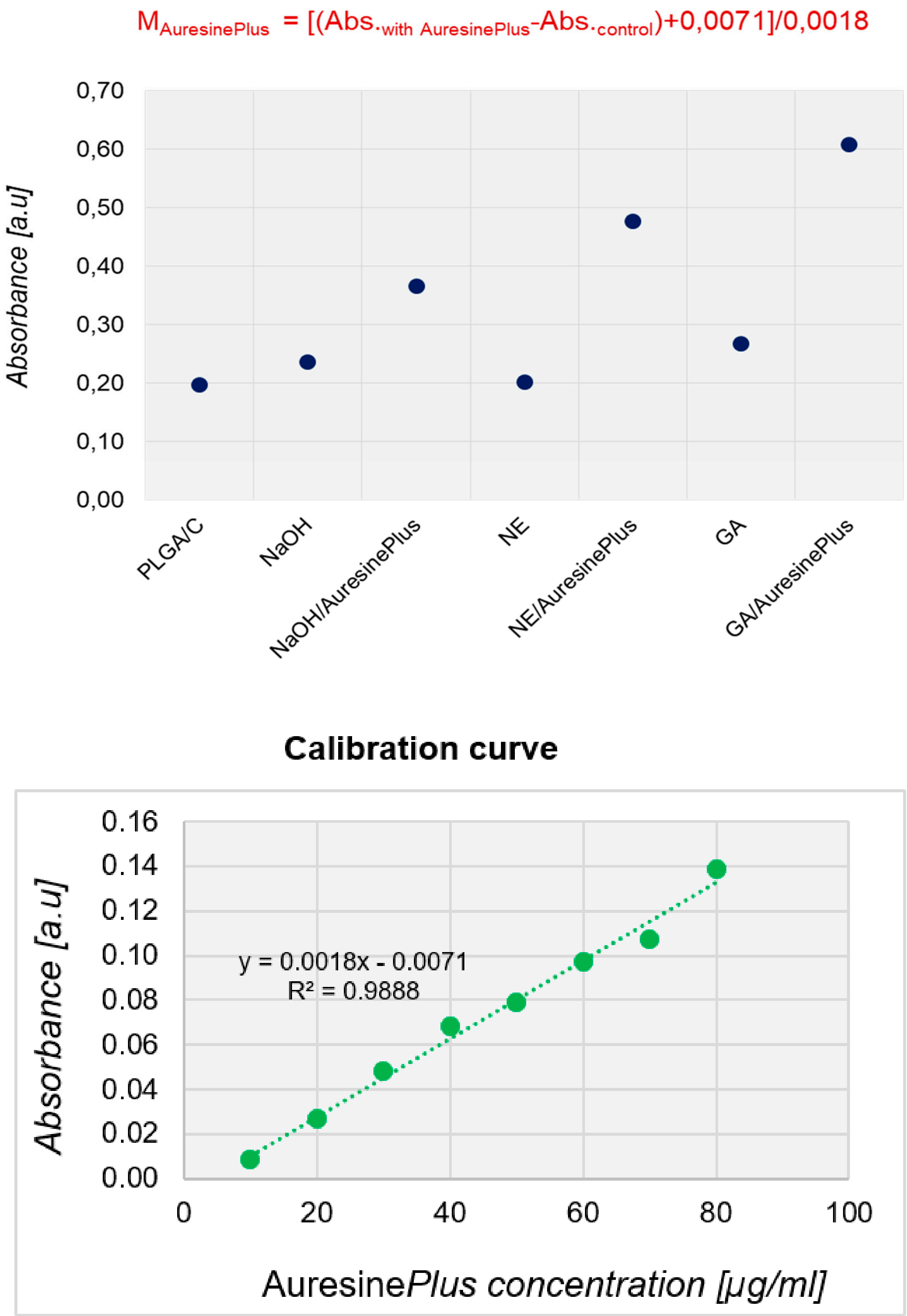

Figure S3. Determination of Auresine*Plus* amount attached to 1 mg of nonvowens. Calculations were done based on BCA assays as described in Material and Methods.

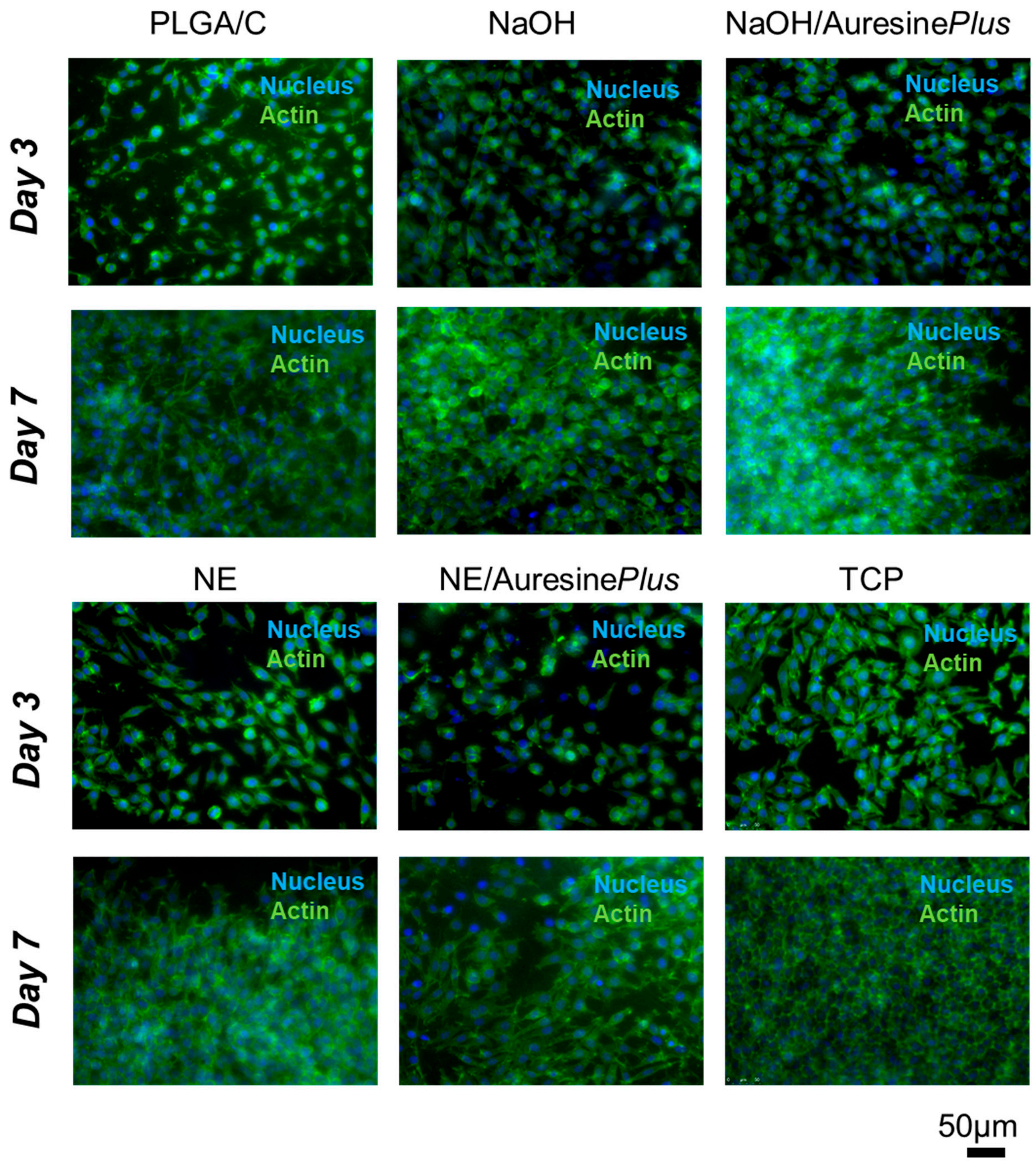

Figure S4. Morphology of fibroblast grown on various fibers and control conditions (TCP).

Table S1. Antimicrobial activity of the functionalized materials – *S.aureus* NCTC 8325-4 eradication upon direct-contact of nonwovens with immobilized Auresine*Plus* and the respective controls. Around 10^6 CFU/ml of bacterial cells in PBS was incubated on the nonwoven surface for 3 hours. Presented values show the number of eradicated bacterial cells in cfu per ml and in relative (%) to the number of bacteria suspended in PBS alone and kept for 3 hours in the same conditions, Arrows indicate decrease (eradication) or **increase** (**growth**) of the bacterial cell number.

**Sample Name**	**NaOH/ AuresinePlus**	**NaOH**	**NE/ AuresinePlus**	**NE**	**GA/ AuresinePlus**	**GA**	**In Solution**	**PLGA/C**
Eradicated Bacteria Assay 1	9.21×10^5 *(99.05%)* ↓	-1.48×10^6 *(−158.66%)* ↑	7.23×10^5 *(77.69%)* ↓	4.18×10^5 *(44.92%)* ↓	-5.83×10^5 *(−62.74%)* ↑	-3.75×10^5 *(−62.74%)* ↑	9.21×10^5 *(99.04%)* ↓	-2.04×10^6 *(−219.61%)* ↑
Eradicated Bacteria, Assay 2	9.23×10^5 *(100.00%)* ↓	1.16×10^5 *(12.58%)* ↓	9.21×10^5 *(99.76%)* ↓	5.79×10^4 *(6.27%)* ↓	-2.52×10^5 *(−27.26%)* ↑	-5.06×10^5 *(−27.26%)* ↑	9.23×10^5 *(100%)* ↓	1.02×10^5 *(11%)* ↓
Average ± SD	$\mathbf{9.22 \times 10^5}$ $\pm 1.52 \times 10^3$ *(100 ± 1%)*	$\mathbf{-6.80 \times 10^5}$ $\pm 1.13 \times 10^6$ *(−73 ± 121%)*	$\mathbf{8.22 \times 10^5}$ $\pm 1.40 \times 10^5$ *(89 ± 16%)*	$\mathbf{2.38 \times 10^5}$ $\pm 2.54 \times 10^5$ *(26 ± 27%)*	$\mathbf{-4.18 \times 10^5}$ $\pm 2.35 \times 10^5$ *(−45 ± 25%)*	$\mathbf{-6.55 \times 10^4}$ $\pm 6.22 \times 10^5$ *(−45 ± 25%)*	$\mathbf{9.22 \times 10^5}$ $\pm 1.60 \times 10^3$ *(100 ± 1%)*	$\mathbf{-9.70 \times 10^5}$ $\pm 1.52 \times 10^6$ *(−104 ± 163%)*
